# A Radiomics Approach to Predicting Parkinson’s Disease by Incorporating Whole-Brain Functional Activity and Gray Matter Structure

**DOI:** 10.3389/fnins.2020.00751

**Published:** 2020-07-15

**Authors:** Xuan Cao, Xiao Wang, Chen Xue, Shaojun Zhang, Qingling Huang, Weiguo Liu

**Affiliations:** ^1^Division of Statistics and Data Science, Department of Mathematical Sciences, University of Cincinnati, Cincinnati, OH, United States; ^2^Department of Radiology, Affiliated Brain Hospital, Nanjing Medical University, Nanjing, China; ^3^Department of Statistics, University of Florida, Gainesville, FL, United States; ^4^Department of Neurology, Affiliated Brain Hospital, Nanjing Medical University, Nanjing, China

**Keywords:** Parkinson’s disease, radiomics, resting-state functional magnetic resonance imaging, structural magnetic resonance imaging, machine learning

## Abstract

Parkinson’s disease (PD) is a progressive, chronic, and neurodegenerative disorder that is primarily diagnosed by clinical examinations and magnetic resonance imaging (MRI). In this study, we proposed a machine learning based radiomics method to predict PD. Fifty healthy controls (HC) along with 70 PD patients underwent resting-state magnetic resonance imaging (rs-fMRI). For all subjects, we extracted five types of 6664 features, including mean amplitude of low-frequency fluctuation (mALFF), mean regional homogeneity (mReHo), resting-state functional connectivity (RSFC), voxel-mirrored homotopic connectivity (VMHC) and gray matter (GM) volume. After conducting dimension reduction utilizing Least absolute shrinkage and selection operator (LASSO), fifty-three radiomic features including 46 RSFCs, 1 mALFF, 3 mReHos, 1 VMHC, 2 GM volumes and 1 clinical factor were retained. The selected features also indicated the most discriminative regions for PD. We further conducted model fitting procedure for classifying subjects in the training set employing random forest and support volume machine (SVM) to evaluate the performance of the two methods. After cross-validation, both methods achieved 100% accuracy and area under curve (AUC) for distinguishing between PD and HC in the training set. In the testing set, SVM performed better than random forest with the accuracy, true positive rate (TPR) and AUC being 85%, 1 and 0.97, respectively. These findings demonstrate the radiomics technique has the potential to support radiological diagnosis and to achieve high classification accuracy for clinical diagnostic systems for patients with PD.

## Introduction

Parkinson’s disease (PD) is a major neurodegenerative disease influenced by both genetic and environmental factors (Halliday et al., 2014). As the second most common neurodegenerative disorder, PD is characterized by the degeneration of dopamine-producing cells in the brain resulting in motor symptoms and nonmotor features (Mhyre et al., 2012). Available diagnostic tools are better at detecting motor symptoms than nonmotor symptoms. The neural and pathophysiologic mechanisms to predict the progression of PD remain unclear and discovering the psychobiological markers is the key research priority. Understanding the inner working mechanisms of PD is one of the most intriguing scientific questions. Studies in neuroscience strongly suggest intervention during early therapeutic windows (Vu et al., 2012; Tibar et al., 2018). Although positron emitted topography/computed tomography is accurate (Meles et al., 2017) the diagnosis of PD at present is mainly dependent on clinical features and scores.

In recent years, neuroimaging has been increasingly employed to aid the early diagnosis of PD. A variety of neuroimaging technologies including functional magnetic resonance imaging (fMRI), structure MRI (sMRI), positron emission tomography (PET) and electroencephalography (EEG) have been widely adopted. Among these, resting-state functional MR imaging (rs-fMRI) is regarded as a promising technique for precisely locating the abnormal spontaneous activities in neuropsychological disease (Wang et al., 2019). Several rs-fMRI based methods including regional homogeneity (ReHo), amplitude of low frequency fluctuations (ALFF), and functional connectivity (FC) provide a task-free approach to explore spontaneous brain activity and connectivity among networks in different brain regions of PD patients. Application of these techniques provides new insights in prediction, early diagnosis and differential diagnosis of PD. Previous rs-fMRI studies using ALFF found specific frequency band of ALFF for PD (Li et al., 2016) and detected significant alterations of ALFF in the prefrontal cortex and subcortical regions in PD patients (Xiang et al., 2016). Frequency domain analyses of ALFF revealed decreased ALFF in the putamen, parieto-temporo-occipital cortex, thalamus, cerebellum, and several occipital regions, while increased ALFF values were detected in the caudate and several temporal regions (Zhang et al., 2013). A large sample study of 109 PD patients found distinguishing frequency bands and neural modulations in the brainstem and striatum correlated with the dose of levodopa and bradykinesia subscale scores (Hou et al., 2014).

Along with these distinctive changes of ALFF, spontaneous ReHo analysis in rs-fMRI studies also achieved considerable progress in examining early onset and late-onset PD (Wu et al., 2009; Yang et al., 2013; Sheng et al., 2016). ReHo alteration in the early phase of PD showed a low level of local coherence in the right primary sensory and positive correlation with disease duration (Choe et al., 2013). A 2-year longitudinal PD study of multimodal MRI using ReHo and voxel-based-morphometry (VBM) observed a progressive decrease of ReHo values in the sensorimotor cortex, default-mode network (DMN), and the left cerebellum, but increased ReHo in the supplementary motor area (SMA), bilateral temporal gyrus, and hippocampus (Zeng et al., 2017). A meta-analysis using ALFF and ReHo found consistent decreased activity in the putamen for PD patients that could serve as an independent validation of rs-fMRI (Wang et al., 2018). Another ALFF and ReHo based study demonstrated the disturbed DMN, SMA, basal ganglia (BG), and posterior cerebellar lobule in cognitively normal PD as compared with healthy controls (Harrington et al., 2017). These multilevel characteristics of rs-fMRI could effectively improve the discrimination accuracy of diagnosis.

Although previous rs-fMRI studies revealed widespread abnormal intrinsic networks in line with the pathophysiology of PD, these findings and biomarkers have not been extensively used for diagnosis, prediction or prognosis of PD in daily clinical practice. In recent years, a method called radiomics that extracts large amount of features from radiographic medical images into high-dimensional mineable data using data-characterization algorithms has received considerable attention, particularly in clinical oncology diagnosis (Valladares et al., 2020). Radiomics analysis employs multimodality medical images and machine learning techniques to extract many quantitative characteristics as objective, sensitive biomarkers of disease stage to potentially detect treatment effects (Liu et al., 2019). Applications of radiomics approach in the neurodegenerative and mental disorder disclosed the heterogeneity characteristics with a high accuracy that facilitate individualized diagnosis in patients with Alzheimer’s disease, autism spectrum disorder and schizophrenia (Hofmann-Apitius et al., 2015; Salvatore et al., 2019; Wang et al., 2019). The radiomics technology that integrated the advantages of various models has been utilized to extract the characteristics for automated diagnosis of early PD and quantifying PD severity. These methods consist of voxel-based method (VBM), diffusion tensor imaging (DTI), functional connectome and connectivity measures among others (Shinde et al., 2019). A radiomics analysis of longitudinal Single-photon Emission Computed Tomography (PSECT) images demonstrated radiomic features significantly increased the prediction accuracy and were proved to be effective prognostic biomarkers of PD (Rahmim et al., 2017). More recently, a radiomics of deep neural nets on neuromelanin-sensitive MRI demonstrated a test accuracy of 85.7% and revealed the substantia nigra pars compacta abnormalities in PD discriminating from atypical PD (Shinde et al., 2019). Another radiomics study of quantitative susceptibility mapping were shown to assist the diagnosis of idiopathic PD (Cheng et al., 2019). A classifier for early PD with an accuracy of 86.96% was identified from SVM training by extracting characteristics including ALFF, ReHo and RSFC from the gray matter (GM), white matter (WM) and cerebrospinal fluid (CSF) (Long et al., 2012).

Considering the above-mentioned radiomics approaches in existing PD studies, we aimed to utilize both rs-fMRI and sMRI to extract radiomic features including whole-brain functional activity (i.e., ALFF and ReHo), connectivity (i.e., RSFC and VMHC) and gray matter (GM). Our goal was to discover more effective biomarkers and to eventually develop an automated classification framework of early diagnosis for PD patients.

## Materials and Methods

### Participates and Clinical Evaluation

This study was approved by the Medical Research Ethical Committee of Nanjing Brain Hospital (Nanjing, China) in accordance with the Declaration of Helsinki, and written informed consent was obtained from all subjects. Seventy PD patients and fifty healthy controls (HC) were recruited. All the demographic characteristics and clinical symptom ratings were collected before MRI scanning and all patients were in the ON state during the MRI scan.

All subjects underwent a complete neurological and psychological status assess, and a review of medical history records. Mini-mental state examination (MMSE) was used to evaluate cognition. The severity of depression was quantified using the Hamilton Depression Scale (HAMD). The neurocognitive tests were administered to each participant individually by a professional appraiser in the neuropsychological research center. All HC participants were interviewed to rule out the presence including current or past psychiatric illness, history of psychiatric illness in first-degree relatives and/or current or past significant medical or neurological illness.

The demographic and clinical data of patients with PD and HC were compared using a Fisher’s exact test (for sex), analysis of variance (ANOVA) (for age, education, MMSE and HAMD). The level of significance was set at *p* < 0.05.

### Image Data Acquisition

Image data were acquired using a Siemens 3.0-Tesla signal scanner (Siemens, Verio, Germany) in the department of radiology within Nanjing Brain Hospital. Functional imaging data were collected transversely by using a gradient-recalled echo-planar imaging (GRE-EPI) pulse sequence with the following configurations: TR/TE = 200 ms/30 ms, flip angle = 90°, matrix = 64 × 64, FOV = 220 × 220 mm, thickness/gap = 3.5/0.6 mm, in-plane resolution = 3.4 × 3.4 mm, slices = 31. For each subject, a total of 140 volumes were obtained, resulting in a total scan time of 280 s. High resolution anatomical images were acquired using a T1 fluid attenuated inversion recovery (FLAIR) sequence (TR/TE = 2530/3.34 ms, flip angle = 7°, matrix = 256 × 192, FOV = 256 × 256 mm, slice thickness/gap = 1.33/0.5 mm, 128 slices covered the whole brain). The subjects were instructed to keep their eyes closed, relax their minds and remain as motionless as possible during the data acquisition. Rubber earplugs were used to reduce noise, and foam cushioning was used to fix the head to reduce motion artifacts. The MR images were retrieved from the archive by two experienced neuroradiologists (QH and XW).

### Data Preprocessing

Image preprocessing procedure was carried out Data Processing Assistant for Resting-State fMRI^1^ based on Statistical Parametric Mapping (SPM12^2^) operated on the Matlab platform. The following steps were applied to the image data. For each subject, we first discarded the first five time points for signal equilibrium when subject was still adapting to the scanning noise. The remaining 135 images underwent slice-timing correction using the middle slice as the reference frame and head motion correction by regressing out 6 head motion signals (displacement on *x*, *y*, and *z* direction and 3 angular motion). Four subjects with more than 2.5 mm maximum displacement in any of the three dimensions or 2.5° of any angular motion were removed. Next, T1-weighted structural images of each subject were coregistrated to the resulting functional images followed by images being segmented into gray matter (GM), white matter, and cerebrospinal fluid using a new segment and DARTEL segmentation algorithm. The functional images were spatially normalized to the Montreal Neurological Institute (MNI) space with 3 × 3 × 3 mm cubic voxels. After normalization, images were spatially smoothed with a 4 mm full width at half maximum (FWHM) Gaussian kernel and detrended using linear, quadratic or higher order polynomial algorithms. Note that when calculating Regional Homogeneity (ReHo), the smoothing procedure was omitted for maintaining the measuring accuracy. After smoothing and detrending, we further regressed out nuisance covariates including the Friston 24 motion parameters (Friston et al., 1996), white matter, global signals and cerebrospinal fluid signals and applied temporal filter (0.01 Hz < f < 0.08 Hz) to diminish high-frequency noise.

### Image Feature Extraction

#### ReHo Analysis

The method of Regional Homogeneity (ReHo) (Zang et al., 2004) was proposed to analyze characteristics of regional brain activity and to reflect the temporal homogeneity of neural activity. It has been pointed out that some preprocessing methods especially spatial smoothing R-fMRI time series may significantly change the ReHo magnitudes (Zuo et al., 2013). To get rid of this potential issue, preprocessed rs-fMRI data without the spatial smoothing step was used for calculating ReHo. All individual ReHo maps were computed and then spatially smoothed with a 4 mm FWHM Gaussian kernel. In particular, we focused on the mReHo maps obtained by dividing the mean ReHo of the whole brain within each voxel in the ReHo map. We further segmented the mReHo maps and extract all the 112 ROI signals based on the Harvard-Oxford atlas (HOA) using the Resting-State fMRI Data Analysis Toolkit, REST^3^.

#### ALFF and VHMC Extraction

Slow fluctuations in activity are fundamental features of the resting brain for determining correlated activity between brain regions and resting state networks. The relative magnitude of these fluctuations can discriminate between brain regions and subjects. Amplitude of Low Frequency Fluctuations (ALFF) (Zang et al., 2007) are related measures that quantify the amplitude of these low frequency oscillations. Leveraging the preprocessed data within the frequency range between 0.01 and 0.1 Hz, we calculated individual ALFF maps and the mALFF maps by dividing the mean ALFF of the whole brain within each voxel in the ALFF maps. Using the HOA, we ended up with 112 mALFF values after extracting the ROI signals based on the mALFF maps.

Voxel-Mirrored Homotopic Connectivity (VMHC) quantifies functional homotopy by providing a voxel-wise measure of connectivity between hemispheres. VMHC calculates the connectivity between each voxel in one hemisphere and its mirrored counterpart in the other (Zuo et al., 2010). By segmenting the VMHC maps via HOA, we also got 112 VHMC values.

#### RSFC and GM Volume Extraction

Resting-state functional connectivity (rsFC) analysis is an effective method for estimating spontaneous functional activity and measuring the temporal correlation between spatially remote neurophysiological events. The preprocessed rs-fMRI images were segmented into 112 ROIs according to HOA. After averaging the rs-fMRI time courses of all the voxels within each ROI, the mean time series of each ROI were acquired. We performed Pearson’s correlation analysis on each pair of ROI time series (i.e., 112 × 111/2 = 6216 pairs in total). The 6216 correlation coefficients were then transformed into z-scores by Fisher’s z transformation and retained as the RSFC metrics. Based on the preprocessed structural images, we also extracted GM volumes of these 112 ROIs using the HOA as masks.

### Feature Selection and Model Validation

Our candidate features consist of all the aforementioned metrics including ReHo, mALFF, VHMC, RSFC and GM volume along with all the clinical characteristics. To build our model, we first randomly split our dataset into training set and testing set while maintaining the PD:HC ratio, where 93 subjects were used as the primary cohort for feature selection and model training. The remaining 23 subjects were treated as validation cohort for examining the selected features. All steps of feature selection and model training were only based on and performed in the training dataset.

Note that our goal was to identify the most significant variables that could discriminate PD patients from healthy controls. However, as we had a total of 6669 features and a comparably much smaller sample size, the dimension reduction was necessary to improve the accuracy in the later step of building the machine learning model for classification (Wang et al., 2019). Hence, we first performed the nonparametric Mann-Whitney U test on each feature between the PD patients and the healthy controls and kept the variables with *P*-value larger than 0.1. Since the Mann–Whitney U test does not require the data to be normally distributed, we adopted the procedure as the first step to filter the features. Next, to avoid the possible presence of Simpson’s Paradox caused by multicollinearity, where a predictor appears to be significant by itself, but this observation disappears or the direction reverses when other predictors are added. Therefore, whenever we spotted an absolute value of pairwise correlation between two features that was larger than 0.5, we removed the feature with larger average absolute correlation. Finally, to further reduce the burden of high dimensionality imposed on the model training, we used the least absolute shrinkage and selection operator (Lasso) procedure that assigns a penalty to the coefficients and eliminates variables with zero coefficient value. We used 10-fold cross validation to obtain the optimal penalty parameter for Lasso and retained the features with nonzero regression coefficients.

**FIGURE 1:**
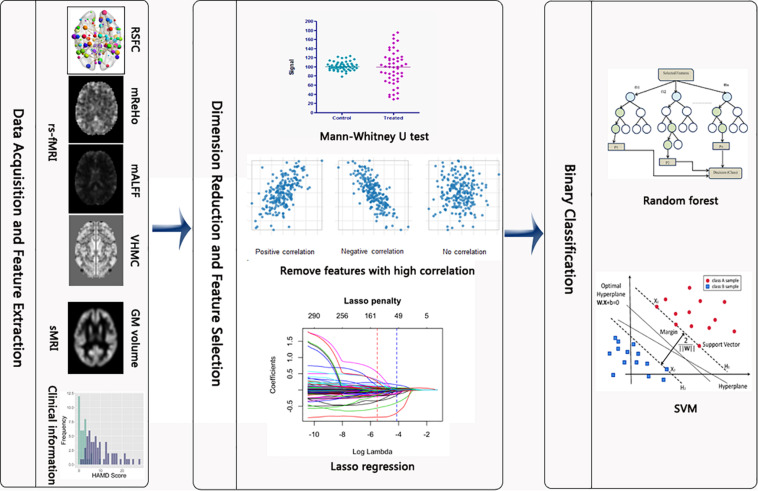
Flowchart of the study. After the rs-fMRI and sMRI images were preprocessed, we extracted the 6669 metrics. Then, Mann–Whitney U test, screening out high correlated variables and Lasso regression were implemented to reduce the number of features. Last, random forest and SVM were conducted to differentiate between PD and HC subjects.

The methods of supper vector machine (SVM) and random forest were implemented for classifying subjects based on the selected features. Given the relatively small sample size, we used the linear kernel when fitting SVM, which was conducted by specifying the type parameter to be “linear” in the svm function. The tuning parameters for both methods were selected via 10-fold cross-validation by using the trainControl function with the method parameter being “repeatedcv” and the train function with the method parameter specified as either “cforest” or “svmLinear2” for random forest and SVM, respectively. The performance of these two different machine learning methods in the training and test sets were later compared and visualized according to different metrics including accuracy, true positive rate, false positive rate, receiver-operating characteristic (ROC) curve and area under curve (AUC). The complete statistical analysis was conducted in R 3.5.0. Specifically, packages “e1071,” “randomForest”, “glmnet,” “caret” were employed for running the SVM, random forest, Lasso and for cross validation, respectively. The flowchart of this study is presented in Figure 1.

## Results

### Clinical Characteristics

In Table 1, we provided the complete demographic and clinical information for all subjects participated in this study. No significant difference was observed with respect to the gender, age, education and MMSE score between PD patients and HCs, while significant difference was detected for HAMD between these two groups. In particular, for PD patients, the HAMD scores (11.0 ± 6.9) were significantly higher than those for HCs (2.1 ± 2.3).

**TABLE 1:** Clinical and demographic data evaluation of PD and HC.

Characteristics	PD (*n* = 68)	HC (*n* = 48)	Test statistics	*P*-value
Sex (M/F)	35/33	23/25	0.409	>0.05^a^
Age (year)	57.8 ± 7.0	57.8 ± 5.5	0.021	>0.05^b^
Education (year)	11.8 ± 3.3	11.7 ± 4.8	0.689	>0.05^c^
MMSE	28.6 ± 1.7	29.0 ± 2.3	0.585	>0.05^d^
HAMD	11.0 ± 6.9	2.1 ± 2.3	67.58	<0.05^e^

*^*a*^The *P*-value for gender distribution by Fisher’s exact test; ^*b*−*e*^The *P*-values for age, education, MMSE and HAMD, respectively, by analysis of variance (ANOVA).*

### Feature Selection

**FIGURE 2:**
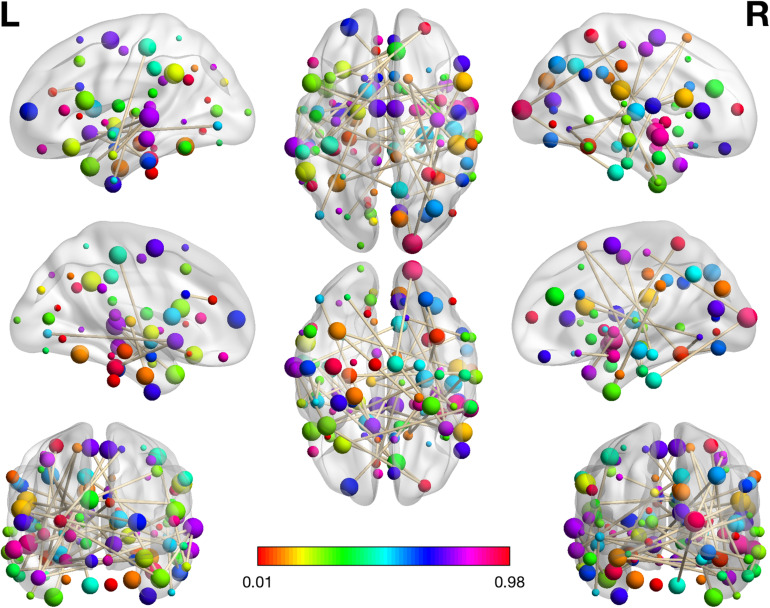
The visualization plot of the selected 46 RSFCs. The color of the spot reflects the number of connections that the associated brain regions participate in. The larger corresponding value on the color bar means more connections are involved in the respective region. The brain networks were visualized with the BrainNet Viewer (Xia et al., 2013).

After the first step of Mann–Whitney U test, 6669 features containing metrics from rs-fMRI, sMRI and clinical information reduced to 993 features. Next, the procedure of excluding variables with absolute correlations larger than 0.5 removed 628 features with a total of 365 features remaining. Last, 54 features including (46 RSFCs, HAMD, 1 mALFF, 3 mReHos, 1 VMHC and 2 GM volumes) with nonzero coefficients obtained from the logistic regression with Lasso penalty were retained as the final metric set to be used for binary classification. In Table 2, we listed these 46 RSFCs and the respective connected brain regions indexed in the HOA template. The related brain regions of RSFCs were primarily located in the executive control network (ECN), default mode network (DMN), affective network (AN), visual network (VIN) and sensorimotor network (SMN) (Figure 2). The seven features were mALFF of the left superior temporal gyrus, posterior division, mReHo of the left parahippocampal gyrus, posterior division, mReHo of the right thalamus and left pallidum, VMHC of the right temporal fusiform cortex, anterior division, and the GM volume of the right inferior temporal gyrus, anterior division and the right accumbens.

**TABLE 2:** 46 RSFC features and the related brain regions indexed in the HOA template.

ID	HOA number	Brain region A	Network	HOA number	Brain region B	Network
1	1	Frontal Pole.L	Other region	86	Parietal Operculum Cortex.R	Other region
2	2	Frontal Pole.R	Other region	5	Superior Frontal Gyrus.L	Other region
3	2	Frontal Pole.R	Other region	15	Temporal Pole.L	AN
4	4	Insular Cortex.R	AN	88	Planum Polare.R	Other region
5	6	Superior Frontal Gyrus.R	Other region	30	Inferior Temporal Gyrus, posterior.R	DMN
6	6	Superior Frontal Gyrus.R	Other region	78	Temporal Occipital Fusiform Cortex.R	VIN
7	7	Middle Frontal Gyrus.L	DMN	56	Paracingulate Gyrus.R	ECN
8	11	Inferior Frontal Gyrus, pars opercularis.L	Other region	76	Temporal Fusiform Cortex, posterior.R	VIN
9	12	Inferior Frontal Gyrus, pars opercularis.R	Other region	72	Lingual Gyrus.R	Other region
10	12	Inferior Frontal Gyrus, pars opercularis.R	Other region	87	Planum Polare.L	Other region
11	14	Precentral Gyrus.R	SMN	70	Parahippocampal Gyrus, posterior.R	DMN
12	15	Temporal Pole.Sup.L	AN	19	Superior Temporal Gyrus, posterior.L	AUN
13	15	Temporal Pole.L	AN	52	Juxtapositional Lobule Cortex.R	Other region
14	16	Temporal Pole.R	AN	36	Superior Parietal Lobule.R	VIN
15	16	Temporal Pole.R	AN	51	Juxtapositional Lobule Cortex.L	Other region
16	16	Temporal Pole.Mid.R.	AN	79	Occipital Fusiform Gyrus.L	VIN
17	17	Superior Temporal Gyrus, anterior.L	DMN	42	Angular Gyrus.R	DMN
18	18	Superior Temporal Gyrus, anterior.R	DMN	104	Right Putamen	BGN
19	19	Superior Temporal Gyrus, posterior.L	DMN	21	Middle Temporal Gyrus, anterior.L	DMN
20	19	Superior Temporal Gyrus, posterior.L	DMN	53	Subcallosal Cortex.L	Other region
21	21	Middle Temporal Gyrus, anterior.L	DMN	33	Postcentral Gyrus.L	SEN
22	22	Middle Temporal Gyrus, anterior.R	DMN	65	Frontal Orbital Cortex.L	Other region
23	23	Middle Temporal Gyrus, posterior.L	DMN	62	Precuneus Cortex.R	DMN
24	24	Middle Temporal Gyrus, posterior.R	DMN	30	Inferior Temporal Gyrus, posterior.R	Other region
25	27	Inferior Temporal Gyrus, anterior.L	DMN	89	Heschl’s Gyrus.L	AUN
26	28	Inferior Temporal Gyrus, anterior.R	DMN	60	Cingulate Gyrus, posterior.R	DMN
27	29	Inferior Temporal Gyrus, posterior.L	DMN	87	Planum Polare.L	Other region
28	32	Inferior Temporal Gyrus, temporooccipital.R	Other region	72	Lingual Gyrus.R	Other region
29	34	Postcentral Gyrus.R	SMN	96	Occipital Pole.R	VIN
30	44	Lateral Occipital Cortex, superior.R	VIN	111	Left Accumbens	Other region
31	45	Lateral Occipital Cortex, inferior.L	VIN	111	Left Accumbens	Other region
32	54	Subcallosal Cortex.R	Other region	110	Right Amygdala	DMN
33	55	Paracingulate Gyrus.L	ECN	57	Cingulate Gyrus, anterior.L	DMN
34	55	Paracingulate Gyrus.L	ECN	100	Right Thalamus	DMN
35	56	Paracingulate Gyrus.R	ECN	85	Parietal Operculum Cortex.L	Other region
36	58	Cingulate Gyrus, anterior.R	ECN	85	Parietal Operculum Cortex.L	Other region
37	58	Cingulate Gyrus, anterior.R	ECN	90	Heschl’s Gyrus.R	AUN
38	60	Cingulate Gyrus, posterior.R	DMN	74	Temporal Fusiform Cortex, anterior.R	VIN
39	70	Parahippocampal Gyrus, posterior.R	Other region	99	Left Thalamus	DMN
40	75	Temporal Fusiform Cortex, posterior.L	VIN	111	Left Accumbens	Other region
41	77	Temporal Occipital Fusiform Cortex.L	VIN	86	Parietal Operculum Cortex.R	Other region
42	77	Temporal Occipital Fusiform Cortex.L	VIN	110	Right Amygdala	DMN
43	78	Temporal Occipital Fusiform Cortex.R	VIN	80	Occipital Fusiform Gyrus.R	VIN
44	88	Planum Polare.R	Other region	99	Left Thalamus	DMN
45	92	Planum Temporale.R	Other region	107	Left Hippocampus	DMN
46	96	Occipital Pole.R	VIN	98	Brainstem.R	Other region

**FIGURE 3:**
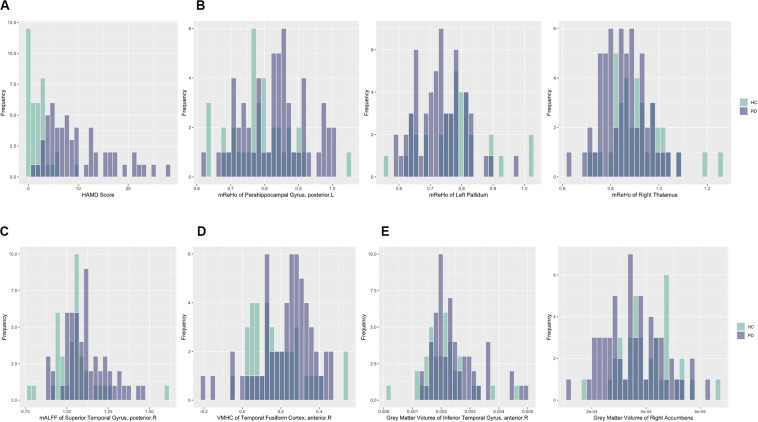
Histograms of selected features for PD and HC subjects with darker color representing overlapping values. Purple: PD; Green: HC. **(A)** HAMD score; **(B)** mReHo values of the left parahippocampal gyrus, posterior division, the right thalamus and left Pallidum; **(C)** mALFF values of the left superior temporal gyrus, posterior division; **(D)** VHMC values of the right temporal fusiform cortex, anterior division; **(E)** GM volumes of the right inferior temporal gyrus, anterior division and the right accumbens.

For better illustration and clearer visualization of the difference for these selected features between PD group and HC group, in Table 3 we reported the mean, standard deviation (SD) and *P*-value of the resulting features from the dimension reduction step for two groups. We also plotted the histograms of these features with different colors representing different groups in Figure 3. In particular, the increasing or decreasing trends of these features between two groups can be immediately revealed by looking at the corresponding values in Table 3 or locations in Figure 3.

**TABLE 3:** The mean, standard deviation (SD) and *P*-value for all 54 selected features in PD group and HC groups.

ID	Feature	PD (mean ± SD)	HC (mean ± SD)	*P*-value
1	Frontal Pole.L-Parietal Operculum Cortex.R	−0.1129 ± 0.2642	−0.2487 ± 0.265	0.3488
2	Frontal Pole.R-Superior Frontal Gyrus.L	0.0478 ± 0.2354	0.0408 ± 0.2536	0.0815
3	Frontal Pole.R-Temporal Pole.L	0.2116 ± 0.2723	0.3172 ± 0.2829	0.2758
4	Insular Cortex.R-Planum Polare.R	0.2713 ± 0.2794	0.1674 ± 0.2358	0.0104
5	Superior Frontal Gyrus.R-Inferior Temporal Gyrus, posterior.R	−0.0828 ± 0.3272	0.0568 ± 0.3013	0.0341
6	Superior Frontal Gyrus.R-Temporal Occipital Fusiform Cortex.R	−0.2821 ± 0.2749	0.4011 ± 0.2316	0.1139
7	Middle Frontal Gyrus.L-Paracingulate Gyrus.R	0.5995 ± 0.2634	0.4751 ± 0.2763	0.0082
8	Inferior Frontal Gyrus, pars opercularis.L-Temporal Fusiform Cortex, posterior.R	0.0897 ± 0.2763	0.2384 ± 0.2609	0.2141
9	Inferior Frontal Gyrus, pars opercularis.R-Lingual Gyrus.R	−0.1656 ± 0.2669	0.2521 ± 0.2320	0.1853
10	Inferior Frontal Gyrus, pars opercularis.R-Planum Polare.L	0.0418 ± 0.2501	0.0845 ± 0.2656	0.0344
11	Precentral Gyrus.R-Parahippocampal Gyrus, posterior.R	0.1467 ± 0.2138	0.0216 ± 0.2398	0.0032
12	Temporal Pole.L-Superior Temporal Gyrus, posterior.L	−0.3148 ± 0.2612	0.4955 ± 0.3027	0.2183
13	Temporal Pole.L-Juxtapositional Lobule Cortex.R	−0.0739 ± 0.2415	0.0455 ± 0.2886	0.0269
14	Temporal Pole.R-Superior Parietal Lobule.R	0.1059 ± 0.2912	0.0107 ± 0.2773	0.0292
15	Temporal Pole.R-Juxtapositional Lobule Cortex.L	0.6623 ± 0.2743	0.7524 ± 0.2624	0.2021
16	Temporal Pole.R-Occipital Fusiform Gyrus.L	−0.1192 ± 0.2378	0.0146 ± 0.2261	0.0045
17	Superior Temporal Gyrus, anterior.L-Angular Gyrus.R	−0.0500 ± 0.2470	0.0688 ± 0.2392	0.0285
18	Superior Temporal Gyrus, anterior.R-Right Putamen	−0.1993 ± 0.2559	0.1071 ± 0.2856	0.0207
19	Superior Temporal Gyrus, posterior.L-Middle Temporal Gyrus, anterior.L	−0.0174 ± 0.2309	0.1275 ± 0.2442	0.1225
20	Superior Temporal Gyrus, posterior.L-Subcallosal Cortex.L	0.0451 ± 0.2116	0.1618 ± 0.2278	0.1429
21	Middle Temporal Gyrus, anterior.L-Postcentral Gyrus.L	−0.0628 ± 0.2827	0.0856 ± 0.2278	0.0130
22	Middle Temporal Gyrus, anterior.R-Frontal Orbital Cortex.L	−0.0029 ± 0.2553	0.1269 ± 0.2147	0.0659
23	Middle Temporal Gyrus, posterior.L-Precuneus Cortex.R	−0.0032 ± 0.1956	0.1271 ± 0.1759	0.0238
24	Middle Temporal Gyrus, posterior.R-Inferior Temporal Gyrus, posterior.R	0.0455 ± 0.2457	0.0399 ± 0.2304	0.0888
25	Inferior Temporal Gyrus, anterior.L-Heschl’s Gyrus.L	0.4837 ± 0.3685	0.3644 ± 0.3915	0.0180
26	Inferior Temporal Gyrus, anterior.R-Cingulate Gyrus, posterior.R	0.1240 ± 0.2235	0.0302 ± 0.3139	0.0049
27	Inferior Temporal Gyrus, posterior.L-Planum Polare.L	−0.0922 ± 0.2427	0.0667 ± 0.2399	0.0035
28	Inferior Temporal Gyrus, temporooccipital.R-Lingual Gyrus.R	−0.0489 ± 0.2319	0.0818 ± 0.1936	0.0103
29	Postcentral Gyrus.R-Occipital Pole.R	0.2362 ± 0.2612	0.1036 ± 0.3113	0.0060
30	Lateral Occipital Cortex, superior.R-Left Accumbens	−0.1134 ± 0.2020	0.0016 ± 0.2128	0.0043
31	Lateral Occipital Cortex, inferior.L-Left Accumbens	0.7454 ± 0.2993	0.6130 ± 0.2801	0.0100
32	Subcallosal Cortex.R-Right Amygdala	−0.1688 ± 0.2078	0.0146 ± 0.2352	0.0006
33	Paracingulate Gyrus.L-Cingulate Gyrus, anterior.L	−0.1056 ± 0.2160	0.0254 ± 0.2486	0.0047
34	Paracingulate Gyrus.L-Right Thalamus	−0.0849 ± 0.2356	0.1805 ± 0.2874	0.2865
35	Paracingulate Gyrus.R-Parietal Operculum Cortex.L	0.0737 ± 0.2734	0.0281 ± 0.2416	0.0475
36	Cingulate Gyrus, anterior.R-Parietal Operculum Cortex.L	0.1262 ± 0.2546	0.2581 ± 0.2542	0.3158
37	Cingulate Gyrus, anterior.R-Heschl’s Gyrus.R	0.0159 ± 0.2817	0.0983 ± 0.1895	0.0704
38	Cingulate Gyrus, posterior.R-Temporal Fusiform Cortex, anterior.R	−0.1340 ± 0.2362	0.0164 ± 0.2544	0.0084
39	Parahippocampal Gyrus, posterior.R-Left Thalamus	0.2082 ± 0.2267	0.3380 ± 0.1966	0.1968
40	Temporal Fusiform Cortex, posterior.L-Left Accumbens	0.1025 ± 0.2688	0.2534 ± 0.2388	0.2285
41	Temporal Occipital Fusiform Cortex.L-Parietal Operculum Cortex.R	0.0059 ± 0.2695	0.0970 ± 0.2492	0.2080
42	Temporal Occipital Fusiform Cortex.L-Right Amygdala	0.1999 ± 0.2317	0.0755 ± 0.2679	0.0043
43	Temporal Occipital Fusiform Cortex.R-Occipital Fusiform Gyrus.R	0.0310 ± 0.2339	0.1158 ± 0.2416	0.2874
44	Planum Polare.R-Left Thalamus	−0.0057 ± 0.2023	0.1249 ± 0.2388	0.0550
45	Planum Temporale.R-Left Hippocampus	−0.1296 ± 0.2257	0.0337 ± 0.2826	0.0017
46	Occipital Pole.R-Brainstem.R	0.0972 ± 0.2042	0.0076 ± 0.2136	0.0096
47	HAMD Score	10.1429 ± 6.3545	1.9730 ± 2.1793	0.0000
48	mALFF of Superior Temporal Gyrus, posterior.R	1.1140 ± 0.1317	1.0478 ± 0.1379	0.0270
49	mReHo of Parahippocampal Gyrus, posterior.L	0.8302 ± 0.0956	0.7870 ± 0.0859	0.0292
50	mReHo of Right Thalamus	0.8533 ± 0.0924	0.9144 ± 0.1154	0.1034
51	mReHo of Left Pallidum	0.7300 ± 0.0772	0.7718 ± 0.1010	0.0910
52	VMHC of Temporal Fusiform Cortex, anterior.R	0.2277 ± 0.1448	0.1818 ± 0.1395	0.0159
53	Gray Matter Volume of Inferior Temporal Gyrus, anterior.R	0.0025 ± 0.0008	0.0021 ± 0.0008	0.0103
54	Gray Matter Volume of Right Accumbens	0.0004 ± 0.0001	0.0004 ± 0.0001	0.1797

### Model Fitting

After the screening process, we were left with only 56 features and were no longer stuck in the ultrahigh dimensional situation. Most of the existing machine learning methods including random forest and SVM could accommodate this relatively smaller number of variables compared with previous 6669 features. Therefore, we conducted the model fitting procedure for classifying subjects in the training set utilizing random forest and SVM to evaluate the performance of these two methods. It turned out that after cross-validation, both random forest and SVM achieved the perfect accuracy and AUC for distinguishing between PD and HC subjects in the training set, which was not surprising since we only had 56 coefficients to estimate while we had a sample size of 93 subjects.

### Model Validation

Despite the superior performance of both methods in the training set, what really matters is the predictive result in the testing set. We therefore examined the validity of random forest and SVM by evaluating their classification performance in the testing set using AUC and the following measures (Table 4),

**TABLE 4:** Predictive performance table in the testing set for random forest and SVM.

	Accuracy	TPR	FPR	AUC
Random forest	0.8261	0.9167	0.2727	0.9015
SVM	0.8483	1	0.3136	0.9697

$$A\,c\,c\,u\,r\,a\,c\,y=\frac{T\,P+T\,N}{T\,P+T\,N+F\,P+F\,N^{\prime }}$$

$$T\,P\,R=\frac{T\,P}{T\,P+F\,N^{\prime }}\,F\,P\,R=\frac{F\,P}{T\,N+F\,P^{\prime }}$$

where TP, TN, FP and FN correspond to true positive, true negative, false positive and false negative, respectively.

**FIGURE 4:**
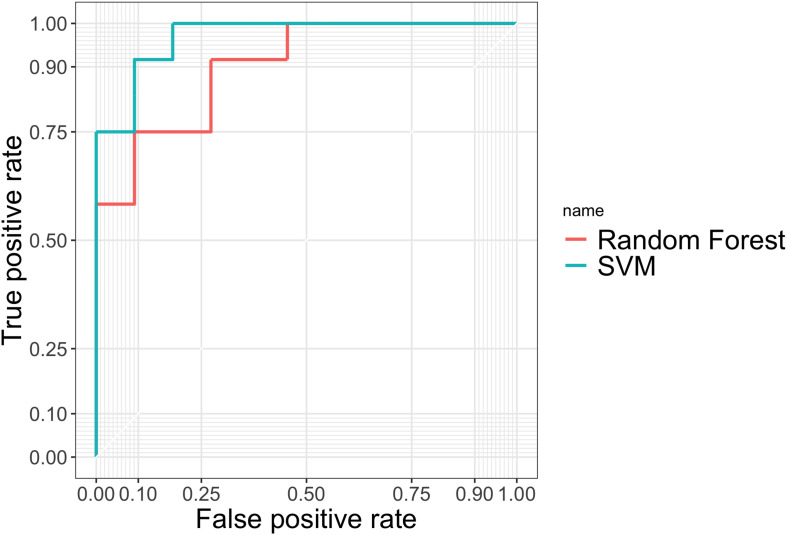
ROC curves evaluating the predictive performance of random forest and SVM in the testing set.

TPR measures the proportion of PD patients correctly detected by the given procedure among all the PD patients. FPR is calculated using the number of people who were falsely identified as having PD, divided by the total number of HCs. Accuracy gives the proportion of true results (both true positives and true negatives) among the total number of cases examined, i.e., the sample size of the testing set. In practice, we used the natural cutoff 0.5 to determine whether the subject should be classified as PD. To further assess the robustness of two methods, we also plotted the ROC curves in Figure 4 by varying thresholding values.

From Table 4 and Figure 4 we can tell that random forest and SVM performed comparably well in terms of the overall accuracy. Random forest performed slightly better than SVM according to FPR, while SVM outperformed random forest according to accuracy, TPR and AUC. Therefore, for a less conservative and more robust prediction, we would prefer SVM over random forest based on the performance summary in the testing set.

## Discussion

We presented a framework for uncovering predictive markers of PD based on radiomics analysis and obtained excellent accuracy in classifying PD from HCs. Our model was established based on the relevant clinical characteristics, whole-brain functional connectivity and activity along with gray matter structure. After collecting the MRI scans for one subject, it may take 2 h for obtaining clinical evaluation, 10 min for preprocessing and around 2 s for running the machine learning models. To the best of our knowledge, this is the first study to explore the whole-brain functional activity and gray matter structure in a homogeneous and relatively large sample MRI study. The distinctive whole-brain functional activity and connection were mainly located within or across the AN, DMN, ECN and SMN in PD compared with HCs.

Our results showed that both methods achieved perfect accuracy in the training set, and SVM yielded an overall better classification performance than random forest in the testing set. In particular, SVM had higher accuracy (85%), TPR (1) and AUC (0.97) than random forest, while the FPR for SVM (0.31) was higher than random forest (0.27). The radiomics-based machine learning models in present study demonstrated the validity of trained classifiers in PD, which could be helpful to support clinical decision in both radiology and neurology.

Previous studies have made great progress in identifying PD and other neurodegenerative disorders from HCs using structural and fMRI data with the assist of machine learning. These results demonstrated the ability of supervised classification methods with a relatively high accuracy. An automatic SVM based study with leap motion controller recruited 16 PD and 12 HCs, and the accuracy was 74.07% with an AUC of 0.675 (Butt et al., 2018). Another study of SPECT imaging using SVM and logistic regression (LR) showed that SVM method produced a higher accuracy of 85% than LR of 83%, and the authors claimed that the SVM-based model could provide better guide for PD stage classification (Hsu et al., 2019). A large sample based on 831 structure T1-weighted MRI achieved a very high accuracy of up to 99% for differential diagnosis of PD (Singh and Samavedham, 2015). A study incorporating DTI and VBM in an SVM algorithm correctly distinguished PD from progressive supranuclear palsy (PSP) when white matter atrophy was considered (Cherubini et al., 2014). Combined with these previous findings, big data-driven approaches were helpful to aid PD diagnosis and to reach precision medicine (Khoury et al., 2019; van den Heuvel et al., 2020).

The aforementioned methods either possessed a lower accuracy and AUC or only considered part of the complete radiomic features based on either rs-MRI or sMRI. In our study, the radiomics approach integrated both rs-MRI and sMRI by extracting features that quantify the whole-brain functional activity and connectivity along with GM volume and clinical evaluations. Forty-six RSFCs and the respective connected brain regions were selected after dimension reduction, and these disturbed brain regions related to RSFCs were primarily located in the ECN, DMN, AN, VN, and SMN. Seven more features of the HAMD, mALFF, mReHo, VMHC and the GM volume were also retained to build the classifying model. Within the model, intrinsic connectivity networks of mALFF were identified and located mainly in DMN including the left superior temporal gyrus, posterior division, the left parahippocampal gyrus, posterior division. The particular neural activities of mReHo maps were also located in DMN of the right thalamus and GN of the left pallidum. Selected VMHC quantified the functional homotopy of connectivity in VN of the right temporal fusiform cortex and anterior division. Features of the GM volume also covered the DMN in the right inferior temporal gyrus, anterior division and the right accumbens.

Some of these findings were expected and in accordance with previous studies. For example, the altered RSFCs were primarily located in the typical resting-state network (RSN). However, the prominent role of DMN, ECN, AN, VIN and sensorimotor functioning in PD revealed in our study was remarkable. The identified regions in DMN included the left superior temporal gyrus, posterior division, the left parahippocampal gyrus, the right thalamus and the right inferior temporal gyrus. We also found abnormal AN in the right insular cortex, left and right temporal pole, along with unusual VIN in lateral occipital cortex, superior, temporal fusiform cortex, the left and right temporal occipital fusiform cortex, the left occipital pole, the right superior parietal lobule. Abnormal ECN was detected in the left and right paracingulate gyrus, and the right anterior cingulate gyrus.

RSN reflects the spontaneous neural activities of the blood oxygenation level-dependent (BOLD) signals between temporally correlated brain regions. Compared with the control group, the DMN plays a crucial role in neurodegenerative disorders and normal aging. Several fMRI studies have indicated that the DMN injured before the cognitive decline in PD (Sandrone and Catani, 2013; Koshimori et al., 2016). A 2-year study using ReHo and VBM to identify differences in local spontaneous brain activity and gray matter volume found that PD patients with normal cognition showed a decreased ReHo in the DMN (Zeng et al., 2017). In addition, a gender-specific effect of uric acid on resting-state cortical FC found the de novo PD group had decreased FC in bilateral cingulate, postcentral and lingual gyri within DMN (Lee et al., 2018). Our results were consistent with these previous studies.

The basal ganglia, thalamus, and brainstem are important in the pathophysiology of PD. Studies on detecting neural activity changes in these regions have achieved more sensitive and reliable results for scientific and clinical research on PD. The basal ganglia network (BGN) has been observed in pathologies with altered neurotransmitter systems of dopaminergic processes, and also involving motor control. In the present study, we found disturbed BGN in left pallidum, the right thalamus and the right brainstem. The variability of FC in healthy older adults found strongest correlate of FC in the BGN, and potential links to dopamine-related function (Griffanti et al., 2018). A sex-related pattern of RSN showed an increased connectivity within the BGN in female PD patients, and FC changes in sensorimotor at baseline were considered as an independent predictor of disease severity in the early stage of PD (De Micco et al., 2019).

Attention networks (AN) in cortical regions are affected in early stage of PD (Madhyastha et al., 2015). Proteinopathy and longitudinal changes in FC networks within the SMN were confirmed, and the interaction between the dorsal attention network (DAN) the frontoparietal control network decreased significantly over time in PD while correlated with the decline in cognitive function (Campbell et al., 2019). Altered organization of the DAN and lack of changes in the ventral attention network (VAN) in PD patients indicated the higher risk for freezing of gait during complex walking situations, and these findings revealed that AN played an important role in freezing of gait (Maidan et al., 2019). Gender-specific effect of uric acid on rs-fMRI networks in de novo PD found decreased FC in bilateral insular, frontal and temporal areas within DAN and bilateral medial temporal and right insular areas within executive control network (ECN) (Lee et al., 2018).

Apart from these RSFC findings, structure MRI has received more research focus on better stability and repeatability compared with rs-fMRI. In our study, GM volume of the right inferior temporal gyrus, anterior division and the right accumbens demonstrated differences in PD as opposed to HC. Atrophy of the putamen and altered FC of the striatal structures in PD revealed key structure-function relationship. Caudate nucleus and putamen atrophy could serve as neuroimaging biomeasures for PD (Owens-Walton et al., 2018). A brain microstructual study found decreased white matter fiber features in the right arcuate fasciculus and bilateral middle cerebellar peduncles. The study also detected increased network connectivity in prodromal early PD, which might indicate the neural compensation (Sanjari et al., 2019). The right accumben as one of selected GM features in the model is an interesting sign. An analysis of dopamine regulation and transporter function found regional brain (the nuclei accumbens, cingulate regions and inferior frontal) were closely related with apathy rating scores and β-amyloidopathy for predicting cognitive decline in advancing PD (Zhou et al., 2020). An event-related fMRI study based on reward-related neural responses showed the left nucleus accumbens with lower activation indicated involvement of the ventral striatum in individuals for further development of PD (Thaler et al., 2019) and PD patients with persistent pain displayed an accumbens-hippocampus disconnection (Polli et al., 2016).

Our study certainly has several limitations. First, although we have carried out detailed clinical evaluation and stage classification for all subjects, due to our limited sample size, we could not further stratify the patients according to the disease severity. Second, RSFCs in the model may be influenced by the different clinical symptoms such as olfaction or depression. Third, cerebellum networks were left out in the model. Although the key molecular events that provoke PD have not been fully understood and the underlying mechanisms involving cerebellum were relatively less reported, considerable evidence has indicated that cerebellum plays an important role in sensorimotor dysregulation and has now received growing attention (Lopez et al., 2020; Maas et al., 2020). For future studies, we will include the cerebellum, and increase our sample size in order to obtain different subgroups based on disease stages.

In agreement with previous rs-fMRI studies, the proposed radiomics method that combined rs-fMRI spontaneous activity, connectivity and structure MRI of gray matter (GM) was proved to be scientifically sound and valid. The machine learning based radiomics approach can help the diagnosis, personalized treatment, and prognosis orientation for patient with PD at a lower cost. This type of radiomics approaches should be widely performed and considered as an automated classification framework for predicting PD in clinical management.

## Data Availability Statement

The datasets generated for this study are available on request to the corresponding author.

## Ethics Statement

The studies involving human participants were reviewed and approved by the Medical Research Ethical Committee of Nanjing Brain Hospital (Nanjing, China). The patients/participants provided their written informed consent to participate in this study.

## Author Contributions

QH and WL conceived and designed the study. QH, XC, XW, SZ, CX, and WL performed the experiments. XC and QH wrote the manuscript. XC, QH, and SZ reviewed and edited the manuscript. All authors read and approved the manuscript.

## Conflict of Interest

The authors declare that the research was conducted in the absence of any commercial or financial relationships that could be construed as a potential conflict of interest.

## Footnotes

1 http://rfmri.org/DPARSF

2 http://www.fil.ion.ucl.ac.uk/spm/

3 http://restfmri.net/forum/index.php?q=rest
